# Fluid Creep as an Independent Predictor of Fluid Overload and Mortality in Critically Ill Patients: A Cohort Study

**DOI:** 10.3390/life15121900

**Published:** 2025-12-12

**Authors:** George Briassoulis, Theodora Antonopoulou, Joanna Velegraki, Stavroula Ilia, Eumorfia Kondili

**Affiliations:** 1 Postgraduate Program “Emergency and Intensive Care in Children, Adolescents and Young Adults”, School of Medicine, University of Crete, 70013 Heraklion, Greece; dwraantonopoulou@gmail.com (T.A.); jvelegraki@gmail.com (J.V.); stavroula.ilia@uoc.gr (S.I.); kondylie@uoc.gr (E.K.); 2 Department of Intensive Care Medicine, University Hospital of Heraklion, 71500 Heraklion, Greece; 3 Pediatric Intensive Care Unit, University Hospital of Heraklion, 71500 Heraklion, Greece

**Keywords:** fluid overload, fluid creep, acute kidney injury, critical care, renal angina index, mortality, de-resuscitation

## Abstract

Background: Fluid overload (FO) is a frequent ICU complication and an important predictor of adverse outcomes. While classically attributed to resuscitative fluids, recent data emphasize the contribution of non-therapeutic “fluid creep” from medication diluents and carrier infusions. This study examined associations between fluid creep, FO, acute kidney injury (AKI), and mortality, and explored the predictive value of the modified Renal Angina Index (mRAI) for AKI risk stratification and FO; Methods: A retrospective cohort of 250 critically ill adults (ICU stay ≥72 h) admitted to a mixed medical–surgical ICU between May 2021 and November 2024 was analyzed. All fluids administered during the first 72 h were categorized and indexed to ideal body weight. Fluid creep included drug diluents, carriers, and flushes. FO% was calculated as [(Cumulative Fluid Balance)/IBW] × 100; Results: Fluid creep was higher in non-survivors (5183 ± 2541 vs. 4354 ± 2171 mL; *p* = 0.008) and correlated with FO, cumulative balance, and total input (r = 0.41 to 0.43; *p* < 0.001). Creep and FO independently predicted ICU mortality. Abnormal mRAI scores were associated with FO and early AKI; Conclusions: Fluid creep and FO were independent mortality predictors. Routine monitoring and minimization of creep, along with structured de-resuscitation protocols, may improve outcomes in critically ill adults.

## 1. Introduction

Intravenous fluid therapy is both lifesaving and potentially harmful in critical illness. It is one of the most frequently administered hospital interventions, particularly in emergency and intensive care settings. While essential for restoring hemodynamic stability, excessive administration predisposes patients to fluid overload (FO)—commonly defined as a >10% increase in body weight—which is consistently associated with impaired oxygenation, organ dysfunction, prolonged ICU stay, and increased mortality [1,2,3].

Fluid overload contributes to tissue edema and organ dysfunction through multiple mechanisms, causes morphological organ damage, and may even be fatal. Cerebral edema may exacerbate delirium and impair cognition; myocardial edema can reduce contractility and impair electrical conduction; pulmonary edema limits gas exchange and decreases compliance, increasing ventilatory support needs; and renal and gastrointestinal edema may impair filtration, tubular reabsorption, and barrier integrity [4,5]. Collectively, these effects underscore FO as a modifiable contributor to ICU morbidity and mortality.

Most studies have focused on total fluid balance without distinguishing between categories of fluid exposure [6]. Increasing evidence highlights the contribution of fluid creep—unintended fluids from drug diluents, carrier solutions, and catheter flushes [7]. Although FO is a well-established risk factor for acute kidney injury (AKI) [8] and prolonged mechanical ventilation [7,9,10], the independent role of fluid creep remains insufficiently defined.

Early identification of patients at risk for AKI is equally important to guide fluid management. The modified Renal Angina Index (mRAI), adapted from pediatric populations, integrates baseline risk factors and early markers of kidney injury into a composite score, improving prediction of severe AKI [11]. Coupling such risk stratification with structured fluid stewardship—the systematic monitoring, rationalization, and reduction of unnecessary fluid exposure—may be critical to mitigating iatrogenic harm from hidden fluid burden.

Pediatric and experimental studies suggest that fluid creep may constitute 30–60% of daily intake and substantially increase sodium and chloride burden [12,13]. In adults, non-resuscitative fluids can account for up to 42% of intake during the first three Intensive Care Unit (ICU) days, and minimizing their use has been associated with reduced FO [14,15]. One large adult study found fluid creep and maintenance fluids to be the predominant contributors to early fluid intake, particularly among mechanically ventilated patients [16]. Despite these signals, the prognostic relevance of fluid creep, independent of total fluid balance, has not been systematically evaluated [17,18].

To address these gaps, we investigated early fluid administration patterns and their associations with fluid overload FO and mortality in critically ill adults, with particular focus on fluid creep as a distinct, potentially modifiable contributor to hidden fluid burden. Exploratory analyses also assessed the correlations of fluid creep with prolonged mechanical ventilation, vasoactive support, severity of illness, and ICU length of stay and the predictive value of the modified Renal Angina Index (mRAI) for early AKI risk stratification and FO.

## 2. Materials and Methods

### 2.1. Study Design and Setting

This retrospective observational cohort study was conducted in the mixed medical–surgical ICU of the University General Hospital of Heraklion, a tertiary academic center in Greece. All adult patients admitted between 1 May 2021 and 30 November 2024, were screened for eligibility. The study was approved by the institutional review board (ID 17747/26-05-2023; ID 21549/18-10-2024/extension), with informed consent waived owing to its retrospective design. All procedures adhered to the Declaration of Helsinki and institutional research guidelines.

### 2.2. Patient Selection

Patients aged 18–80 years who remained in the ICU for at least 72 h were eligible for inclusion. Exclusion criteria comprised pre-existing chronic kidney disease, severe heart failure requiring fluid restriction, end-of-life palliative care, and incomplete critical data (i.e., missing essential clinical or laboratory parameters).

### 2.3. Data Collection

Data were extracted from electronic health records, including: Demographics: age, sex, body weight, and height (used to calculate ideal body weight [IBW] for normalization across sex and BMI categories). Admission characteristics: primary diagnosis, pre-admission data, comorbidities, admission type (medical/surgical). Severity indices: Acute Physiology and Chronic Health Evaluation II (APACHE II), Sequential Organ Failure Assessment (SOFA), modified Renal Angina Index (mRAI), Kidney Disease: Improving Global Outcomes (KDIGO) stage. Laboratory/clinical data: admission and daily measurements for 72 h. Interventions: mechanical ventilation, vasopressors, CRRT, nephrotoxic/contrast exposure.

### 2.4. Fluid Data

All fluids administered during the first 72 h were quantified and indexed to IBW. Categories included fluid creep (drug diluents, carrier infusions, flushes), maintenance, resuscitation (≥10 mL/kg crystalloids), replacement, nutrition, and blood products. Solutions were classified as balanced crystalloids or 0.9% sodium chloride (NS). Medication concentrations were maximized per protocol to reduce fluid burden. The handling of continuously administered medications was standardized by means of AI-driven algorithms embedded within the electronic database, which calculated the type and minimum volume of diluents required according to the prescribed dose and infusion rate. All fluid data were automatically extracted from the electronic medication administration records (recorded in real time during infusions) and were not calculated manually. Resuscitation fluids, maintenance fluids, blood products, and nutritional support were administered in accordance with current guidelines specific to each critical illness [19,20,21]. Fluid balance was calculated daily and cumulatively as Fluid Balance (L) = Total Input (L) − Total Output (L). Fluid overload percentage (FO%) was defined using the formula [22] FO% = [(Cumulative Fluid Balance in L)/IBW in kg] × 100. FO > 10% of IBW defined clinically significant FO, and FO > 15% defined severe FO.

### 2.5. Acute Kidney Injury and mRAI

AKI was defined using KDIGO criteria, with severe AKI defined as stage ≥2 at admission or on day 3. Renal function was monitored daily (serum creatinine, urea, estimated creatinine clearance). The mRAI was calculated within 12 h of admission by multiplying risk and injury domains [23,24]. A score ≥6 indicated high risk for severe AKI. A schematic of the scoring system is provided in Figure S1.

### 2.6. Statistical Analysis

Analyses were performed with SPSS v30 (IBM, Armonk, NY, USA). Continuous variables were tested for normality (Shapiro–Wilk) and expressed as mean ± SD or median (IQR). Categorical variables were reported as *n* (%). Between-group comparisons used ANOVA or Kruskal–Wallis tests for continuous variables and chi-square or Fisher’s exact tests for categorical variables. Correlations between fluid balance, fluid overload (FO), and individual fluid categories with illness severity, mRAI, and other clinical variables were assessed using Pearson’s correlation coefficient. Multivariable logistic regression (backward stepwise) was used to identify independent predictors of ICU mortality, FO > 10%, and severe AKI. All clinically relevant covariates (APACHE II, SOFA, KDIGO day-3 stage ≥ 2, mRAI, mechanical ventilation, vasopressor use, age, sex, and fluid variables) were included as candidate predictors. Univariate binary logistic regressions identified individual predictors. Variables with *p* < 0.10 or clinical relevance were subsequently included in a multivariable model. A backward stepwise likelihood-ratio (BSTEP-LR) model was used to derive the most parsimonious set of predictors. Collinearity was assessed via variance inflation factors (VIF). Model discrimination and calibration were evaluated using the area under the ROC curve (AUC), Brier score, Cox & Snell R^2^, Nagelkerke R^2^, and the Hosmer–Lemeshow goodness-of-fit test. Predictive performance of fluid creep, input, FO%, and fluid balance for ICU mortality was evaluated using ROC analysis. Significance was set at *p* < 0.05.

## 3. Results

### 3.1. Demographic and Clinical Characteristics

Between May 2021 and November 2024, 2000 patients were admitted to the ICU. After exclusions, 250 patients had complete datasets (Figure S2). Of these, 160 (64%) were male and 90 (36%) females, with a mean age of 64.8 ± 17 years, body weight 80.1 ± 17 kg, BMI 28.2 ± 5.6, and admission APACHE II score 21.9 ± 7.8. Mean ICU and hospital stays were 15.1 ± 16 and 35.6 ± 31 days, respectively (Table 1).

Non-survivors had lower body weight (*p* = 0.006), higher SOFA scores (*p* = 0.032), lower GCS (*p* = 0.049), and were more often admitted for medical indications (72.2% vs. 56.7%, *p* = 0.020). ICU-acquired infections and infections at admission were also more common in non-survivors (both *p* < 0.05). ICU mortality was 20%; mortality among enrolled patients was 31.6%, and hospital mortality was 47.8%. Non-survivors required more vasoactive days (*p* < 0.001) and had higher rates of mechanical ventilation and vasopressor use (both *p* < 0.001).

### 3.2. Laboratory Findings

On day 3, non-survivors had lower pH (*p* = 0.042), bicarbonate (*p* = 0.009), base excess (*p* = 0.040), and PaO_2_ (*p* = 0.025), with persistently higher lactate from day 2 onward (*p* < 0.001) (Table S1). Longitudinally, sodium, chloride, and potassium increased (all *p* < 0.001), while glucose and lactate declined in survivors (*p* < 0.001). Arterial blood gases improved from day 1 except for PaO_2_, which decreased (*p* < 0.001). Sodium and chloride were not associated with fluid creep, FO%, input, creatinine clearance, or outcomes.

### 3.3. Acute Kidney Injury

At admission, 24.5% had KDIGO stage 2 and 26.6% stage 3 AKI. By day 3, severe AKI (KDIGO ≥ 2) decreased among survivors (14.6%) but rose in non-survivors (27.7%, *p* = 0.011). By day 7, the gap widened (12.8% vs. 32.0%, *p* < 0.001) (Table 2). Creatinine clearance improved in survivors (*p* < 0.001) but not in non-survivors. CRRT use was higher among non-survivors (34.2% vs. 14.8%, *p* < 0.001). Nephrotoxic drug and diuretic exposure did not differ.

Abnormal mRAI (≥6) within 12 h was more common in ICU non-survivors (32.9% vs. 21.1%, *p* = 0.044) and hospital non-survivors (31.9% vs. 18.5%, *p* = 0.014). mRAI independently predicted severe AKI in medical (OR 0.93, 95% CI 0.87–0.99, *p* = 0.032) and emergency patients (OR 0.94, 95% CI 0.88–0.99, *p* = 0.023).

### 3.4. Categories of Administered Fluids

#### 3.4.1. Pre-ICU

Among 73 patients with data, mean intake was 1589 ± 1351 mL crystalloids (25 ± 22 mL/kg IBW) and 957 ± 123 mL blood products/albumin (15 ± 14 mL/kg IBW), with no subgroup differences.

#### 3.4.2. ICU Days 1–3

During the first three ICU days, fluid creep and maintenance fluids each contributed >40% of total intake, while resuscitation and replacement accounted for ~14% and nutrition/blood products ~5% (Table S2, Figure S3). Indexed to IBW, boluses, resuscitation fluids, albumin, and blood products decreased over time, whereas maintenance, creep, and nutrition increased (Table S3).

**Figure 1:**
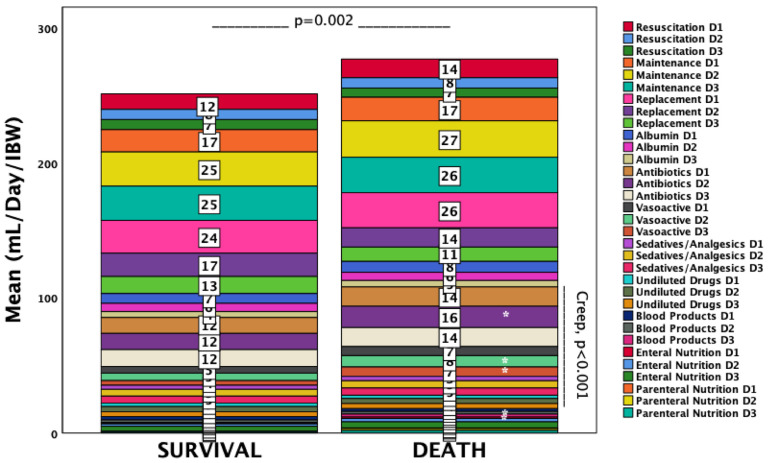
Distribution of daily fluid input by category through ICU day 3, indexed to ideal body weight (IBW) and stratified by survival status. Non-survivors received higher proportions of fluid creep, blood products (day 3), and enteral nutrition (day 1). * *p* < 0.05.

Cumulative fluid creep was higher in non-survivors (5183 ± 2541 vs. 4354 ± 2171 mL, *p* = 0.008), a difference persisting when nutrition and blood products were included (“fluid creep plus”: 6342 ± 3044 vs. 5158 ± 2565 mL, *p* = 0.002; “fluid creep plus indexed to IBW”: 100.7 ± 44.6 vs. 78.3 ± 37.9 mL/IBW, *p* < 0.001) (Table S4). Among input categories, antibiotic diluents (day 2), vasoactive infusions (days 2–3), blood products (day 3), and enteral nutrition (day 1) were significantly higher in non-survivors (all *p* < 0.05) (Figure 1).

**Figure 2:**
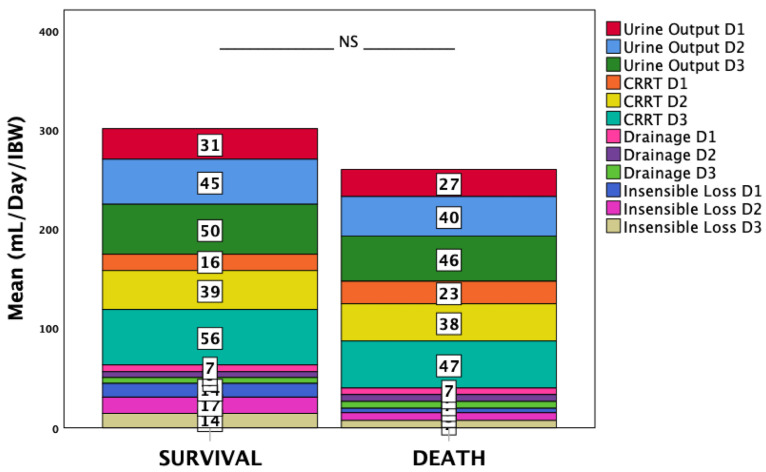
Distribution of daily fluid output by category through ICU day 3, indexed to ideal body weight (IBW) and stratified by survival status.

Other intake categories did not differ; therefore, overall input was greater in non-survivors (*p* = 0.002). Daily and cumulative output volumes were lower in non-survivors, though differences did not reach significance (Figure 2).

### 3.5. Fluid Types

Before ICU, PlasmaLyte and Ringer’s Lactate predominated. During ICU stay, boluses were mostly NS (56–62% day 1, 80–90% days 2–3). Maintenance fluids were largely Ringer’s Lactate with 5–20% D/W. Creep consisted mainly of NS, D/W 5%, and sterile water, used as diluents. Overall composition (~50% balanced crystalloids, 25% NS, 25% D/W/nutrition/blood products) did not differ between survivors and non-survivors.

### 3.6. Fluid Overload

By day 3, clinically significant FO (>10%) and severe FO (>15%) were both significantly more frequent in non-survivors (34.2% and 15.2%, respectively; both *p* < 0.001) (Table 3). Non-survivors also had higher cumulative fluid creep, input, and positive balances.

**Figure 3:**
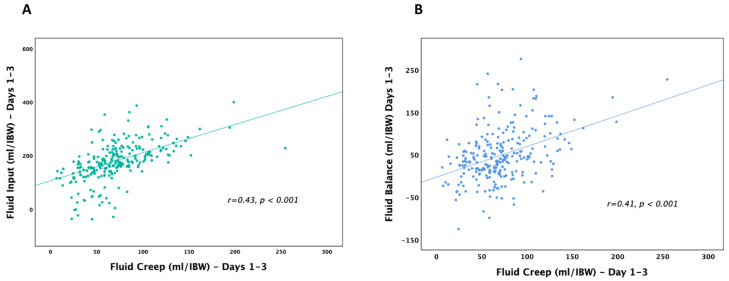
Correlation of cumulative fluid creep with (**A**) total input and (**B**) fluid balance through ICU Day 3, both indexed to IBW. Fluid creep correlated strongly with intake and fluid balance and more weakly with output, underscoring its role in fluid accumulation.

Cumulative fluid creep correlated with FO (r = 0.41, *p* < 0.001), balance (r = 0.41, *p* < 0.001), and input (r = 0.43, *p* < 0.001), and more weakly with mRAI score (r = 0.17, *p* = 0.008) and output (r = 0.17, *p* = 0.006) (Figure 3). It was also associated with longer ICU stay, prolonged mechanical ventilation, and increased vasoactive days (all *p* < 0.001). Cumulative fluid balance was positively correlated with cumulative resuscitation (r = 0.21, *p* < 0.001) and maintenance fluids (r = 0.20, *p* = 0.002) on day 3, as well as with the mRAI score (r = 0.257, *p* < 0.001) and the markers of illness severity including APACHE II (r = 0.30, *p* < 0.001) and SOFA score (r = 0.15, *p* = 0.016). Among all fluid categories, only cumulative resuscitation fluid demonstrated a significant association with SOFA score (r = 0.205, *p* = 0.002). No meaningful correlations were observed between fluid exposure variables and ICU length of stay, duration of mechanical ventilation, or duration of vasopressor support.

In logistic regression, only APACHE II (OR 1.14, 95% CI 1.06–1.22, *p* < 0.001), mRAI (OR 1.08, 95% CI 1.03–1.14, *p* = 0.004), cumulative fluid creep (OR 1.04, 95% CI 1.02–1.06, *p* < 0.001), and maintenance (OR 1.03, 95% CI 1.01–1.05, *p* = 0.005), but not resuscitation fluid volume, independently predicted FO > 10%. For FO > 15%, the same predictors remained significant except for maintenance. The model demonstrated very good explanatory performance (Cox & Snell R^2^ = 0.293; Nagelkerke R^2^ = 0.469) and excellent calibration (Hosmer–Lemeshow χ^2^ = 3.33–3.35; *p* ≈ 0.91). Classification accuracy was ~85% overall (96% for non-FO patients; 36% for FO > 10%), and no problematic multicollinearity was detected (all VIF < 5).

### 3.7. Outcomes

On univariate logistic regression, cumulative fluid creep on day 3 indexed to IBW, total FO% on day 3, total fluid input, KDIGO day-3 stage ≥ 2, and SOFA score were significantly associated with ICU mortality (Table S5). When all clinically relevant variables were entered simultaneously in a multivariable forced-entry model (Table S6), only cumulative fluid creep (aOR 1.017, 95% CI 1.003–1.030; *p* = 0.017) and FO (aOR 1.062, 95% CI 1.001–1.126; *p* = 0.046) remained independently associated with mortality. Inclusion of MV and vasopressor support attenuated the significance of other predictors, consistent with their interdependence with fluid balance and illness severity. Model performance was acceptable (AUC 0.71; Brier 0.19). In the primary backward stepwise likelihood-ratio model (BSTEP-LR; Table S7), the same two variables, fluid creep (OR 1.02, 95% CI 1.01–1.03, *p* = 0.012) and FO (OR 1.06, 95% CI 1.007–1.13, *p* = 0.028), were retained as independent predictors with superior discrimination (AUC 0.95; Brier 0.07), moderate explanatory power (Nagelkerke R^2^ = 0.229; Cox & Snell R^2^ = 0.164), and good calibration (Hosmer–Lemeshow *p* = 0.121). Multicollinearity was assessed via variance inflation factors (all VIF < 5).

**Figure 4:**
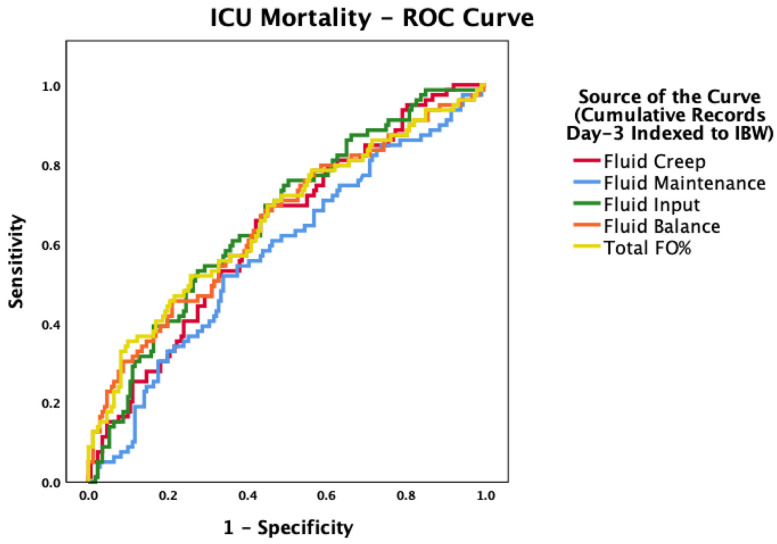
Receiver operating characteristic (ROC) curves for cumulative fluid indices indexed to IBW in predicting ICU mortality. Total input, FO%, fluid balance, maintenance fluids, and cumulative fluid creep showed modest but significant discriminatory ability.

ROC analysis confirmed predictive value for input (AUC 0.674, 95% CI 0.594–0.754, *p* < 0.001), FO% (0.636, 95% CI 0.547–0.725, *p* = 0.003), fluid balance (0.636, 95% CI 0.547–0.725, *p* = 0.003), maintenance fluids (0.625), 95% CI 0.539–0.710, *p* = 0.004, and creep (0.623, 95% CI 0.539–0.707, *p* = 0.004) (Figure 4). Detailed cutoff and sensitivity/specificity data are provided in the Supplement (Table S8).

## 4. Discussion

In this retrospective adult ICU cohort, early fluid creep—unintended fluids from drug diluents, carrier solutions, and catheter flushes—was independently associated with FO and ICU mortality, even after adjusting for illness severity, conventional fluid categories, and early AKI markers. Fluid creep also correlated with prolonged mechanical ventilation, vasoactive support, and ICU length of stay, supporting a pathophysiologic role of hidden fluid burden in promoting interstitial edema and organ dysfunction.

Fluid accumulation contributes to organ dysfunction through multiple mechanisms and can lead to structural tissue injury and death, reinforcing FO as a modifiable driver of morbidity in the ICU. Cerebral edema from excess fluid may exacerbate delirium and impair cognition; myocardial edema can reduce contractility and precipitate conduction disturbances and diastolic dysfunction; pulmonary edema compromises gas exchange, reduces compliance, and increases ventilatory support needs; and renal or gastrointestinal edema may impair filtration, tubular reabsorption, nutrient absorption, and mucosal barrier integrity. These pathophysiological effects support the observed associations between FO and adverse outcomes and underscore the importance of targeted fluid stewardship [4,5].

These findings align with prior reports showing that maintenance fluids and fluid creep often exceed resuscitative boluses in their contribution to daily fluid, sodium, and chloride loads. In pediatric cohorts, higher intake—rather than reduced output—was the main driver of FO, which was associated with longer ICU stay, higher AKI incidence, fewer ventilator-free days, and increased mortality [2,25,26,27]. In a large mixed ICU cohort, creep accounted for ~33% of daily intake—fivefold greater than resuscitation fluids—and was a major source of electrolyte burden [13,28]. Likewise, among patients receiving respiratory support, fluid creep represented ~25% of intake within 24 h and persisted in those with severe hypoxemia [16]. Our results extend these observations by demonstrating that fluid creep is not only common but also prognostically relevant in critically ill adults.

In our analysis, cumulative fluid creep independently predicted clinically significant FO (>10%) and severe FO (>15%), both of which were associated with mortality. These findings reinforce that fluid creep—rather than resuscitation fluids—is the primary driver of early fluid accumulation leading to clinically significant FO in this mixed-ICU cohort. Notably, both fluid creep and FO independently predicted ICU death. These findings highlight fluid creep as a modifiable early exposure, reinforcing the rationale for targeted interventions such as optimizing drug concentrations, minimizing carrier volumes, and preferentially using balanced diluents [15]. The SALT-ED and SMART trials support this biological plausibility, demonstrating reduced kidney injury with balanced fluids compared with saline [29]. Moreover, structured fluid stewardship—progressing through resuscitation, optimization, stabilization, and de-escalation phases—provides a framework to individualize volume targets, prevent FO, and improve outcomes [30].

Consistent with broader ICU evidence, FO in our study was associated with impaired creatinine clearance and increased use of CRRT among non-survivors [31,32]. Notably, abnormal mRAI scores within the first 12 h were more frequent in non-survivors and independently predicted severe AKI, particularly in medical and emergency subgroups. These results align with previous validation studies showing that the mRAI surpasses serum creatinine changes alone in identifying patients at risk for AKI progression [11,31,32]. Although initially validated in pediatric populations [33,34], emerging data support its adaptation for adult and cardiac ICU cohorts, demonstrating comparable predictive accuracy for AKI and other adverse renal outcomes. In critically ill adults, mRAI scores modified by substituting the transplantation variable with diabetes or sepsis, when determined within the first 24 h of ICU admission, outperformed early serum creatinine changes in predicting the development of AKI Stage ≥ 2 during days 2–7 of ICU stay [11]. Furthermore, the RAI has been identified as a strong predictor not only of severe AKI but also of long-term adverse outcomes, enhancing 12-month risk stratification among cardiac ICU patients [35]. While exploratory, our findings support the potential integration of the mRAI into early fluid stewardship protocols to identify high-risk patients for whom minimizing fluid creep and maintenance fluid exposure may be most beneficial [23,35].

Fluid composition also matters. A meta-analysis of five ICU studies (*n* = 1105) found that balanced crystalloids, when used for creep and maintenance, significantly reduced daily sodium burden compared with saline [36]. Similarly, a prospective before–after study showed that substituting 5% glucose for maintenance and diluent fluids halved daily sodium load and lowered daily fluid balance [17]. In our cohort, predominant use of balanced crystalloids, maintained acid–base homeostasis and preserved normal sodium and chloride levels, with no association between electrolyte trends and outcomes. In line with this, a randomized controlled trial found higher chloride concentrations with saline compared with balanced crystalloids (111 vs. 108 mmol/L) but no difference in AKI or mortality [37]. A recent systematic review, however, suggested that balanced crystalloids probably reduce 90-day mortality compared with saline, underscoring the importance of fluid type even in hidden sources such as diluents [38].

From a clinical perspective, our results argue for explicit accounting of fluid creep in early ICU care and its incorporation into fluid stewardship bundles. Practical measures include daily reporting of creep volumes, use of concentrated infusions, balanced diluents when compatible, and integration with the “four Ds” (drug, dosing, duration, de-escalation) [39,40]. Equally important, structured deresuscitation protocols—emphasizing active fluid removal with diuretics or timely initiation of renal replacement therapy—are a complementary step to prevent progression from clinically significant to severe FO and thereby reduce morbidity and mortality [41,42,43]. Although our ROC analyses showed only modest discrimination (AUC 0.62–0.67), creep- and FO-related variables may still function as operational triggers to reassess volume status, initiate deresuscitation, or minimize creep exposure, rather than as strict prognostic thresholds.

This study systematically disaggregated fluid categories indexed to IBW, evaluated early exposures, and linked creep to patient-centered outcomes while adjusting for illness severity and early AKI indices [13]. Limitations include its retrospective, single-center design, potential residual confounding from illness severity and medication burden, and restricted ability to evaluate the impact of fluid type. We acknowledge that detailed data on pre-ICU fluid administration were unavailable, which may have influenced early fluid balance through perioperative fluid delivery, variable equilibration of the interstitial fluid compartment following excess pre-admission intake, fluid redistribution, and potential disease-specific effects on fluid handling [5]. Finally, mRAI analyses were exploratory, not powered for mortality prediction, and ROC performance was modest.

Given the rapidly expanding role of artificial intelligence in critical care, emerging machine-learning approaches, embedded within human-centered clinical systems and fostered through transdisciplinary collaboration, may leverage high-dimensional data on patient characteristics, hemodynamics, and fluid exposure to personalize fluid therapy and enable earlier prediction of fluid overload risk. Such tools have the potential to enhance situational awareness, support timely decision-making, and ultimately improve patient outcomes [44].

## 5. Conclusions

Fluid creep is an underrecognized but modifiable driver of fluid overload and ICU mortality. Incorporating its measurement and reduction into fluid stewardship programs offers a pragmatic strategy to minimize unintended harm. Prospective trials—testing concentration protocols, balanced diluents, and automated monitoring—are warranted to determine whether reducing hidden fluid exposure can improve outcomes in critically ill patients.

## Figures and Tables

**Table 1:** Demographic and clinical characteristics of the study population stratified by survival status.

Patient Characteristics	Total	Survival	Death	*p*-Value
Patients, *n* (%)	250 (100)	171 (68.4)	79 (31.6)	
Gender, *n* (%)				0.115
• Male	160 (64.0)	115 (67.3)	45 (57.0)	
• Female	90 (36.0)	56 (32.7)	34 (43.0)	
Age (years), mean ± SD	64.8 ± 17	63.6 ± 17	67.5 ± 16	0.081
Body weight (kg), mean ± SD	80.1 ± 17	82.1 ± 17	75.8 ± 16	0.006
BMI (kg/m^2^), mean ± SD	28.2 ± 5.6	28.6 ± 5.6	27.4 ± 5.6	0.124
BMI Nutritional Status, *n* (%)				0.156
• Undernutrition (<18.5 kg/m^2^)	8 (3.2)	7 (4.1)	1 (1.3)	
• Normal weight (18.5–25 kg/m^2^)	118 (47.2)	73 (42.7)	45 (57.0)	
• Overweight (25–30 kg/m^2^)	93 (37.2)	69 (40.4)	24 (30.4)	
• Obesity (>25 kg/m^2^)	31 (12.4)	22 (12.9)	9 (11.4)	
APACHE II score, mean ± SD	21.9 ± 7.8	21.1 ± 7.5	23.8 ± 8.4	0.068
SOFA score, mean ± SD	8.45 ± 7.8	8.18 ± 2.9	9.05 ± 3.0	0.032
Glascow Coma Scale, mean ± SD	6.6 ± 4.9	7.0 ± 5.2	5.7 ± 4.2	0.049
Comorbidities, *n* (%)	231 (92.4)	156 (91.2)	75 (94.9)	0.560
Admission Type, *n* (%)				0.020
Medical, *n* (%)	154 (61.6)	97 (56.7)	57 (72.2)	
Surgical, *n* (%)	96 (38.4)	74 (43.3)	22 (27.8)	
Nosocomial Infection, *n* (%)	89 (35.6)	49 (28.7)	40 (50.6)	<0.001
Primary Clinical Diagnoses				0.125
Respiratory	90 (36.0)	55 (32.2)	35 (44.3)	
Cardiac	8 (3.2)	6 (3.5)	2 (2.5)	
Sepsis/Septic Shock	47 (18.8)	27 (15.8)	20 (25.3)	
Traumatic Brain Injury	17 (6.8)	13 (7.6)	4 (5.1)	
Surgery	43 (17.2)	35 (20.5)	8 (10.1)	
Neurological	21 (8.4)	17 (9.9)	4 (5.1)	
Other	19 (7.6)	14 (8.2)	5 (6.3)	
Admission day worst value				
Heart rate, mean ± SD	80.1 ± 32	78.6 ± 30	83.5 ± 35	0.253
Respiratory rate, mean ± SD	26.3 ± 5.8	26.2 ± 31	26.6 ± 6.1	0.566
Systolic blood pressure, mean ± SD	115 ± 29.3	114 ± 27.7	115 ± 32	0.740
Diastolic blood pressure, mean ± SD	58.4 ± 15	59.0 ± 15	57.1 ± 15	0.343
Mean Blood Pressure, mean ± SD	77.1 ± 17	77.5 ± 17	76.3 ± 19	0.612
SpO_2_ (%), mean ± SD	94.6 ± 4.5	94.9 ± 4.3	94.1 ± 4.9	0.179
Therapeutic Interventions, *n* (%)				
• Mechanical ventilation	97 (39.6)	28 (16.9)	69 (87.3)	<0.001
• Vasoactive agents	71 (28.5)	10 (5.9)	61 (77.2)	<0.001
ICU stay (days), mean ± SD	15.1 ± 16	14.2 ± 16	16.9 ± 16	0.220
Hospital stays (days), mean ± SD	35.6 ± 31	39.3 ± 31	27.7 ± 29	0.006
Mechanical ventilation (days), mean ± SD	14.5 ± 16	13.3 ± 16	16.9 ± 16	0.098
Vasoactive therapy (days), mean ± SD	11.8 ± 12	9.9 ± 11	15.5 ± 14	<0.001

Abbreviations: BMI, body mass index; SOFA, Sequential Organ Failure Assessment; APACHE II, Acute Physiology and Chronic Health Evaluation II; ICU, intensive care unit; SpO_2_, oxygen saturation.

**Table 2:** Distribution of acute kidney injury (AKI) markers, treatments, and nephrotoxic exposures by outcome group.

Clinical Characteristics	Total	Survivors	Non-Survivors	*p*-Value
Patients, *n* (%)	250 (100)	171 (68.4)	79 (31.6)	
Urea D1 (mg/dL), mean ± SD	65.4 ± 48	61.4 ± 48	73.9 ± 48	0.056
Urea D2 (mg/dL), mean ± SD	59.1 ± 40 *	55.2 ± 40 *	67.5 ± 39	0.023
Urea D3 (mg/dL), mean ± SD	51.5 ± 33 *^,^**	47.1 ± 30 *^,^**	60.9 ± 36 *	0.0092
Creatinine D1 (mg/dL), mean ± SD	1.42 ± 1.2	1.40 ± 1.3	1.44 ± 1.1	0.836
Creatinine D2 (mg/dL), mean ± SD	1.36 ± 1.0	1.33 ± 0.9	1.44 ± 1.0	0.421
Creatinine D3 (mg/dL), mean ± SD	1.22 ± 0.7 *^,^**	1.17 ± 0.7 *^,^**	1.33 ± 0.8 **	0.107
ClCr D1 (mL/min/1.73 m^2^), mean ± SD	51.7 ± 37	53.5 ± 37	47.6 ± 37	0.244
ClCr D2 (mL/min/1.73 m^2^), mean ± SD	51.9 ± 38	54.2 ± 37	47.1 ± 39	0.173
ClCr D3 (mL/min/1.73 m^2^), mean ± SD	55.0 ± 39 *^,^**	57.8 ± 38 *^,^**	49.1 ± 40	0.101
AKI at admission, *n* (%)				
KDIGO 1	46 (48.9)	28 (50.9)	18 (46.2)	0.901
KDIGO 2	23 (24.5)	13 (23.6)	10 (25.6)	
KDIGO 3	25 (26.6)	14 (25.5)	11 (28.2)	
KDIGO ≥ 2 (day 3), *n* (%)	52 (20.8)	19 (14.6)	33 (27.7)	0.011
KDIGO ≥ 2 (day 7), *n* (%)	48 (21.8)	15 (12.8)	33 (32.0)	<0.001
mRAI (score), mean ± SD	7.1 ± 7.8	6.4 ± 7.1	8.6 ± 8.9	0.043
mRAI (6–40), *n* (%)	62 (24.8)	36 (21.1)	26 (32.9)	0.044
Nephrotoxicity, *n* (%)				0.164
Nephrotoxic (NF) Drugs	49 (19.6)	40 (23.4)	9 (11.4)	
Contrast Agents (CA)	80 (32)	51 (29.8)	29 (36.7)	
AC and NF drugs	31 (12.4)	20 (11.7)	11 (13.9)	
None	90 (36)	60 (35.1)	30 (38.0)	
Diuretics, *n* (%)	184 (93.9)	134 (92.4)	50 (98.0)	0.150
CRRT, *n* (%)	59 (24.1)	19 (14.8)	40 (34.2)	<0.001
Diuretics, (days), mean ± SD	11.1 ± 14	10.7 ± 15	11.9 ± 13	0.559
CRRT, (days), mean ± SD	10.5 ± 10	8.7 ± 7.4	12.9 ± 13	0.201

Abbreviations: AKI, acute kidney injury; KDIGO, Kidney Disease: Improving Global Outcomes; ClCr, creatinine clearance; CRRT, continuous renal replacement therapy; mRAI, modified Renal Angina Index. Related-Samples Friedman’s Two-Way Analysis of Variance by Ranks, *p* < 0.05: * D2 or D3 vs. D1, ** D2 vs. D3.

**Table 3:** Cumulative input, output, fluid balance, and fluid overload by ICU Day 3, stratified by survival status.

Clinical Characteristics	Total	Survivors	Non-Survivors	*p*-Value
Patients, *n* (%)	250 (100)	171 (68.4)	79 (31.6)	
Total Input (mL), mean ± SD	11,561 ± 5366	11,024 ± 5943	12,724 ± 3599	0.020
Total Input (mL/IBW), mean ± SD	180.7 ± 85.5	169.3 ± 91.5	205.5 ± 64.8	0.002
Total Output (mL), mean ± SD	9627 ± 4226	10,007 ± 4426	8804 ± 3649	0.036
Total Output (mL/IBW), mean ± SD	148.9 ± 60.3	152.7 ± 62.0	140.5 ± 54.1	0.137
Fluid Balance (mL), mean ± SD	3038 ± 3877	2327 ± 3298	4577 ± 4556	<0.001
Fluid Balance (mL/IBW), mean ± SD	48.8 ± 62.0	36.6 ± 52.1	75.1 ± 73.1	<0.001
FO%, mean ± SD	4.9 ± 6.2	3.7 ± 5.2	7.5 ± 7.3	<0.001
FO > 10% (clinically significant), *n* (%)	43 (17.2)	16 (9.4)	27 (34.2)	<0.001
FO > 15% (severe), (%)	17 (6.8)	5 (2.9)	12 (15.2)	<0.001

Abbreviations: FO, fluid overload; IBW, ideal body weight. Clinically significant FO was defined as >10% IBW, and severe FO as >15% IBW.

## Data Availability

The datasets used and/or analysed during the current study are available from the corresponding author on reasonable request.

## Supplementary Materials

The following supporting information can be downloaded at: https://www.mdpi.com/article/10.3390/life15121900/s1, Figure S1: Modified Renal Angina Index (mRAI). The mRAI was calculated 12 h after ICU admission as the product of risk and injury domains. The creatinine score was defined as the difference between serum creatinine at ICU admission and the most recent value within the preceding 3 days. An mRAI score ≥6 indicated high risk for acute kidney injury (AKI); Figure S2: Flowchart of patient screening, exclusions, and final study cohort, according to STROBE recommendations; Table S1: Laboratory Tests During the First Three Days of ICU Stay; Table S2: Distribution of daily fluid intake categories stratified by outcome category; Table S3: Distribution of daily fluid intake indexed by ideal body weight (IBW), stratified by outcome category; Figure S3: Distribution of cumulative fluid input categories by ICU day 3, indexed to IBW. Fluid creep and maintenance fluids each accounted for >40% of total intake, whereas resuscitation, replacement, and nutrition/blood products contributed smaller proportions; Table S4: Distribution of cumulative fluid categories by outcome category; Supplementary Methods: Supplementary logistic regression analyses were conducted to assess the robustness of associations between fluid variables and ICU mortality. Univariate binary logistic regressions identified individual predictors (Table S5). Variables with *p* < 0.10 or clinical relevance were subsequently included in a multivariable forced-entry model (Table S6). A backward stepwise likelihood-ratio (BSTEP-LR) model was used to derive the most parsimonious set of predictors (Table S7). Collinearity was assessed via variance inflation factors (VIF > 5 = moderate; VIF > 10 = high). Model discrimination and calibration were evaluated using the area under the ROC curve (AUC), Brier score, Cox & Snell R^2^, Nagelkerke R^2^, and the Hosmer–Lemeshow goodness-of-fit test. Analyses were performed in SPSS v30 (IBM Corp.) and verified in Python 3.12 (statsmodels v0.14); Table S5: Univariate Logistic Regression for ICU Mortality. Selected variables significantly associated with ICU mortality in univariate logistic regression; Table S6: Multivariable Logistic Regression (Forced-Entry Model); Table S7: Backward Stepwise (Likelihood-Ratio) Logistic Regression; Table S8: Predictive ability of cumulative fluid variables by day 3, indexed to ideal body weight (IBW) for ICU mortality, based on ROC analysis.

## Abbreviations

The following abbreviations are used in this manuscript:

AKI	Acute Kidney Injury
FO	Fluid Overload
ICU	Intensive Care Unit
mRAI	modified Renal Angina Index
IBM	Ideal Body Weight
