# Supplementary material for: Fluid Creep as an Independent Predictor of Fluid Overload and Mortality in Critically Ill Patients: A Cohort Study
Source: Life (Basel). 2025 Dec 12;15(12):1900. doi: 10.3390/life15121900 (PMC12735204; doi:10.3390/life15121900)
Supplement: Supplementary file 1 [file life-15-01900-s001.zip › life-3950399-supplementary.pdf]

Elevated Creatinine	Score		Condition	Score	
<0.1 mg/dl	1		ICU Admission	1	
≥0.1 mg/dl	2	X	Diabetes Melitus	3	= mRAI Score
≥0.3 mg/dl	4		Vasopressor or Ventilator	5	
≥0.4 mg/dl	8				

Figure S1. Modified Renal Angina Index (mRAI). The mRAI was calculated 12 hours after ICU admission as the product of risk and injury domains. The creatinine score was defined as the difference between serum creatinine at ICU admission and the most recent value within the preceding 3 days. An mRAI score ≥ 6 indicated high risk for acute kidney injury (AKI).

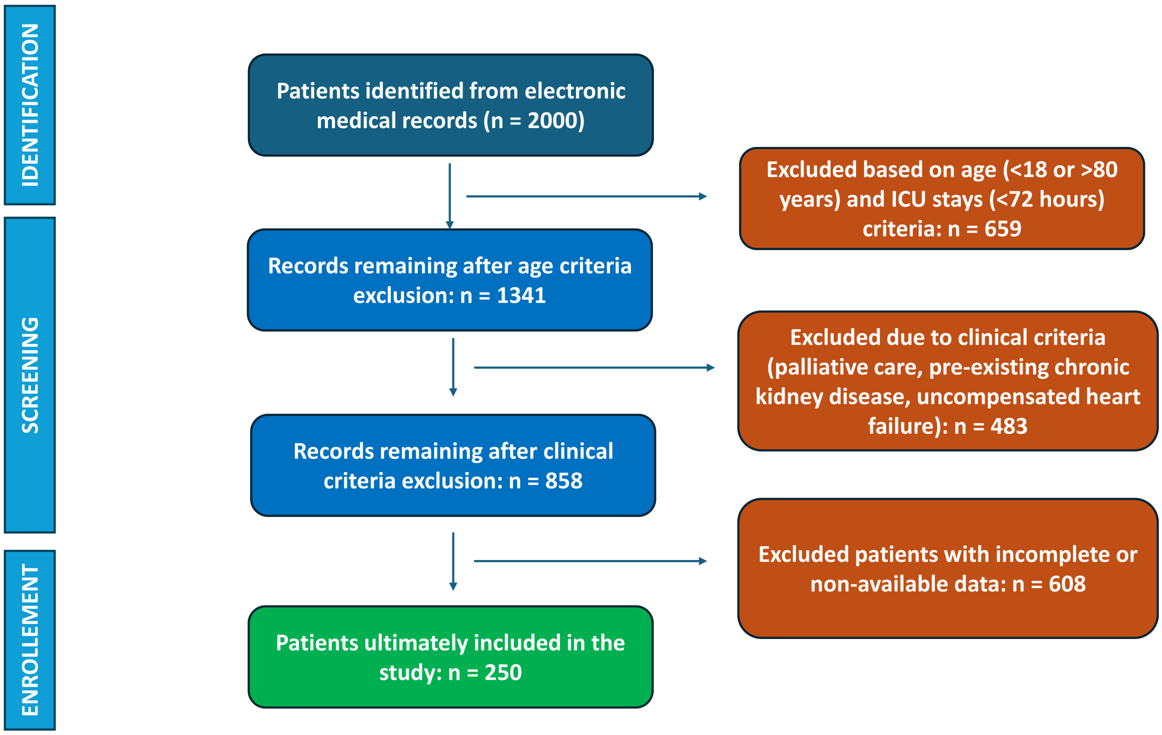

Figure S2. Flowchart of patient screening, exclusions, and final study cohort, according to STROBE recommendations.

Table S1. Laboratory Tests During the First Three Days of ICU Stay

Laboratory Results (Days 1-3)	Total	Survival	Death	p-value
**Patients, n (%)**	250 (100)	171 (68.4)	79 (31.6)	
**pH D1, mean±SD**	7.32 ± 0.11	7.32 ± 0.11	7.31 ± 0.13	0.373
**pH D2, mean±SD**	7.36 ± 0.03 *	7.37 ± 0.07 *	7.35 ± 0.09 *	0.129
**pH D3, mean±SD**	7.39 ± 0.07 *,**	7.39 ± 0.07 *,**	7.37 ± 0.08 *	0.042
**PaO_2 (mmHg) D1, mean±SD**	109 ± 57	110 ± 56	108 ± 62	0.785
**PaO_2 (mmHg) D2, mean±SD**	88.7 ± 29 *	88.9 ± 28 *	88.3 ± 31	0.852
**PaO_2 (mmHg) D3, mean±SD**	79.2 ± 29 *	81.9 ± 28 *	73.2 ± 19 *	0.025
**$PaCO_2$ (mmHg) D1, mean±SD**	44.4 ± 1 3	44.2 ± 12	44.8 ± 14	0.710
**$PaCO_2$ (mmHg) D2, mean±SD**	43.6 ± 31 *	44.4 ± 37	41.9 ± 9.8 *	0.562
**$PaCO_2$ (mmHg) D3, mean±SD**	41.7 ± 9.0 *	41.9 ± 8.8 *	41.2 ± 9.4 *	0.567
**HCO_3 (mEq/L) D1, mean±SD**	22.1 ± 6.0	22.2 ± 5.4	21.9 ± 7.2	0.696
**HCO_3 (mEq/L) D2, mean±SD**	23.4 ± 5.3	23.8 ± 4.8 *	22.7 ± 6.2	0.123
**HCO_3 (mEq/L) D3, mean±SD**	24.9 ± 4.8 *,**	25.5 ± 4.4 *,**	23.8 ± 5.5 *,**	0.009
**BE (mmol/L) D1, mean±SD**	-3.7 ± 6.2	-3.6 ± 5 .7	-4.0 ± 7.2	0.664
**BE (mmol/L) D2, mean±SD**	-2.0 ± 5.3 *	-1.8 ± 4.7 *	-2.4 ± 6.3	0.397
**BE (mmol/L) D3, mean±SD**	-0.5±5.3 *,**	-0.01±4.7 *,**	-1.5±6.6 *,**	0.040
**Na D1 (mEq/L), mean±SD**	139 ± 6.1	139 ± 5.9	140 ± 6.2	0.204
**Na D2 (mEq/L), mean±SD**	142 ± 5.7 *	142 ± 5.8 *	143 ± 6.1 *	0.629
**Na D3 (mEq/L), mean±SD**	143 ± 5.5 *	143 ± 5.5 *	143 ± 6.1 *	0.412
**Cl D1 (mEq/L), mean±SD**	104 ± 6.6	104 ± 6.7	104 ± 6.7	0.826
**Cl D2 (mEq/L), mean±SD**	108 ± 7.1 *	108 ± 7.2 *	107 ± 7.7 *	0.359
**Cl D3 (mEq/L), mean±SD**	108 ± 9.2 *	109 ± 8.6 *	107 ± 10.4 *	0.056
**K D1 (mEq/L), mean±SD**	4.3 ± 0.7	4.3 ± 0.7	4.3 ± 0.7	0.708
**K D2 (mEq/L), mean±SD**	4.5 ± 0.5 *	4.5 ± 0.5 *	4.5 ± 0.5 *	0.780
**K D3 (mEq/L), mean±SD**	4.5 ± 0.4 *	4.5 ± 0.4 *	4.6 ± 0.5 *	0.528
**Glucose D1 (mg/dL), mean±SD**	177 ± 76	179 ± 78	173 ± 72	0.598
**Glucose D2 (mg/dL), mean±SD**	170 ± 60	172 ± 59	167 ± 62	0.565
**Glucose D3 (mg/dL), mean±SD**	148 ± 42 *,**	146 ± 41 *,**	153 ± 45	0.210
**Lactate D1 (mg/dL), mean±SD**	29.8 ± 21	28.3 ± 19	33.1 ± 24	0.088
**Lactate D2 (mg/dL), mean±SD**	25.9 ± 21*	22.5 ± 19 *	33.3 ± 23	<0.001
**Lactate D3 (mg/dL), mean±SD**	20.8 ± 16 *,**	17.7 ± 11 *,**	27.5 ± 21.1 *,**	<0.001

*PaO_2, Partial Pressure of Oxygen; $PaCO_2$, Partial Pressure of Carbon Dioxide; BE, Base Excess. Related-Samples Friedman's Two-Way Analysis of Variance by Ranks, $p<0.05$: *D2 or D3 vs. D1, ** D2 vs. D3.*

Table S2. Distribution of daily fluid intake categories stratified by outcome category.

Daily Fluid Categories	Total	Survivors	Non-survivors	p-Value
**Patients, n (%)**	250 (100)	171 (68.4)	79 (31.6)	
**Fluids (ml/day)**		mean±SD		
**Resuscitation Day 1**	807 ± 785	776 ± 794	878 ± 785	0.388
**Resuscitation Day 2**	482 ± 356	486 ± 117	474 ± 431	0.854
**Resuscitation Day 3**	453 ± 312	476 ± 308	399 ± 317	0.186
**Maintenance Day 1**	1081 ± 664	1076 **± 717**	1092 ± 535	0.854
**Maintenance Day 2**	1637 ± 553 *	1622 ± 551 *	1669 ± 559 *	0.534
**Maintenance Day 3**	1620 ± 524 *	1624 ± 513 *	1613 ± 549 *	0.880
**Replacement Day 1**	1498± 1691	1475 ± 1915	1498 ± 1127	0.948
**Replacement Day 2**	1029 ± 1781	1128 ± 2105	803 ± 509	0.433
**Replacement Day 3**	739 ± 475	819 ± 499	587 ± 391 *	0.062
**Albumin Day 1**	493 ± 302	482 ± 293	517 ± 325	0.609
**Albumin Day 2**	387 ± 268	391 ± 326	382 ± 185	0.904
**Albumin Day 3**	298 ± 308	291 ± 368	308 ± 219	0.884
**Fluid Creep Day 1**	1409 ± 1034	1343 ± 1007	1552 ± 1084	0.138
**Fluid Creep Day 2**	1678 ± 842 *	1563 ± 728 *	1928 ± 1009 *	0.001
**Fluid Creep Day 3**	1528 ± 745 *,**	1448 ± 725 **	1702 ± 763 **	0.012
**Blood Products Day 1**	153.4 ± 450	166.5 ± 497	125.1 ± 330	0.500
**Blood Products Day 2**	118.5 ± 371	108.7 ± 351	139.6 ± 412	0.542
**Blood Products Day 3**	83.44 ± 272	57.8 ± 203	138.8 ± 376	0.028
**Enteral Nutrition Day 1**	36.4 ± 168	20.2 ± 113	71.5 ± 247	0.025
**Enteral Nutrition Day 2**	133.4 ± 282	121.5 ± 254	159.1 ± 336	0.329
**Enteral Nutrition Day 3**	250.1 ± 370 *	230.3 ± 323	292.9 ± 456	0.215
**Parenteral Nutrition Day 1**	13.7 ± 83.2	7.25 ± 55.0	27.6 ± 123	0.072
**Parenteral Nutrition Day 2**	46.9 ± 233	28.7 ± 189	86.2 ± 304	0.070
**Parenteral Nutrition Day 3**	80.5 ± 288 *	63.1 ± 260	118.0 ± 339	0.161
**Fluid Creep Plus* Day 1**	1613 ± 1214	1537 ± 1185	1776 ± 1267	0.148
**Fluid Creep Plus* Day 2**	1977 ± 1021 *	1822 ± 900 *	2313 ± 1182 *	<0.001
**Fluid Creep Plus* Day 3**	1942 ± 963 *	1799 ± 893 *	2252 ± 1038 *	<0.001

*Related-Samples Friedman's Two-Way Analysis of Variance by Ranks, p<0.05: *D2 or D3 vs. D1, ** D2 vs. D3. [a] Fluid Creep Plus = Fluid Creep including Blood Products, and Nutrition.*

Table S3. Distribution of daily fluid intake indexed by ideal body weight (IBW), stratified by outcome category.

Daily Fluid Categories	Total	Survivors	Non-survivors	p-Value
**Patients, n (%)**	250 (100)	171 (68.4)	79 (31.6)	
**Fluids (ml/IBW/day)**		mean±SD		
**Resuscitation Day 1**	12.3 ± 11.5	12.3 ± 11.5	13.9 ± 11.9	0.182
**Resuscitation Day 2**	7.56 ± 4.48	7.54 ± 4.71	7.62 ± 6.89	0.938
**Resuscitation Day 3**	7.28 ± 4.76	7.46 ± 4.84	6.85 ± 5.39	0.485
**Maintenance Day 1**	16.9 ± 11.1	16.6 ± 12.3	17.5 ± 8.1	0.556
**Maintenance Day 2**	25.9 ± 10.0 *	25.3 ± 10.1 *	27.1 ± 9.7 *	0.201
**Maintenance Day 3**	25.7 ± 10.2 *	25.5 ± 10.1 *	26.3 ± 10.2 *	0.535
**Replacement Day 1**	24.9 ± 29.2	24.4 ± 32.9	26.1 ± 10.2	0.771
**Replacement Day 2**	16.4 ± 24.2	17.3 ± 28.2	14.3 ± 10.8	0.598
**Replacement Day 3**	11.9 ± 8.4	12.6 ± 7.4	10.6 ± 10.1 *	0.361
**Albumin Day 1**	7.5 ± 4.9	7.3 ± 4.8	8.1 ± 5.4	0.443
**Albumin Day 2**	6.2 ± 4.9	6.2 ± 5.6	6.1 ± 3.3	0.985
**Albumin Day 3**	4.5 ± 4.7	4.4 ± 5.6 *	4.7 ± 3.3	0.868
**Fluid Creep Day 1**	21.6 ± 15.1	20.3 ± 24.3	24.3 ± 15.8	0.050
**Fluid Creep Day 2**	26.1 ± 13.1 *	23.8 ± 11.0 *	31.0 ± 15.8 *	<0.001
**Fluid Creep Day 3**	23.7 ± 11.2 *,**	21.9 ± 15.1 **	27.3 ± 11.8 **	<0.001
**Blood Products Day 1**	2.38 ± 7.01	2.55 ± 7.63	2.01 ± 5.47	0.572
**Blood Products Day 2**	1.86 ± 6.12	1.73 ± 6.25	2.12 ± 5.85	0.643
**Blood Products Day 3**	1.23 ± 3.77	0.83 ± 2.83	2.08 ± 5.18	0.015
**Enteral Nutrition Day 1**	0.53 ± 2.41	0.29 ± 1.59	1.04 ± 3.54	0.020
**Enteral Nutrition Day 2**	2.03 ± 4.28 *	1.80 ± 3.81 *	2.52 ± 5.17	0.218
**Enteral Nutrition Day 3**	3.81 ± 5.63 *,**	3.47 ± 4.89 *,**	4.52 ± 6.94 *	0.172
**Parenteral Nutrition Day 1**	0.23 ± 1.38	0.11 ± 0.85	0.47 ± 2.11	0.054
**Parenteral Nutrition Day 2**	0.74 ± 3.62	0.44 ± 2.82	1.38 ± 4.88	0.055
**Parenteral Nutrition Day 3**	1.26 ± 4.45 *	0.97 ± 3.89	1.91 ± 5.45	0.122
**Fluid Creep Plus[a] Day 1**	24.7 ± 17.9	23.2 ± 17.5	27.8 ± 18.5	0.058
**Fluid Creep Plus[a] Day 2**	30.7 ± 15.9 *	27.8 ± 13.9 *	37.1 ± 18.1 *	<0.001
**Fluid Creep Plus[a] Day 3**	29.9 ± 14.4 *	27.3 ± 13.1 *	35.8 ± 15.3 *	<0.001

*Related-Samples Friedman's Two-Way Analysis of Variance by Ranks, p<0.05: *D2 or D3 vs. D1, ** D2 vs. D3. [a] Fluid Creep Plus = Fluid Creep including Blood Products, and Nutrition.*

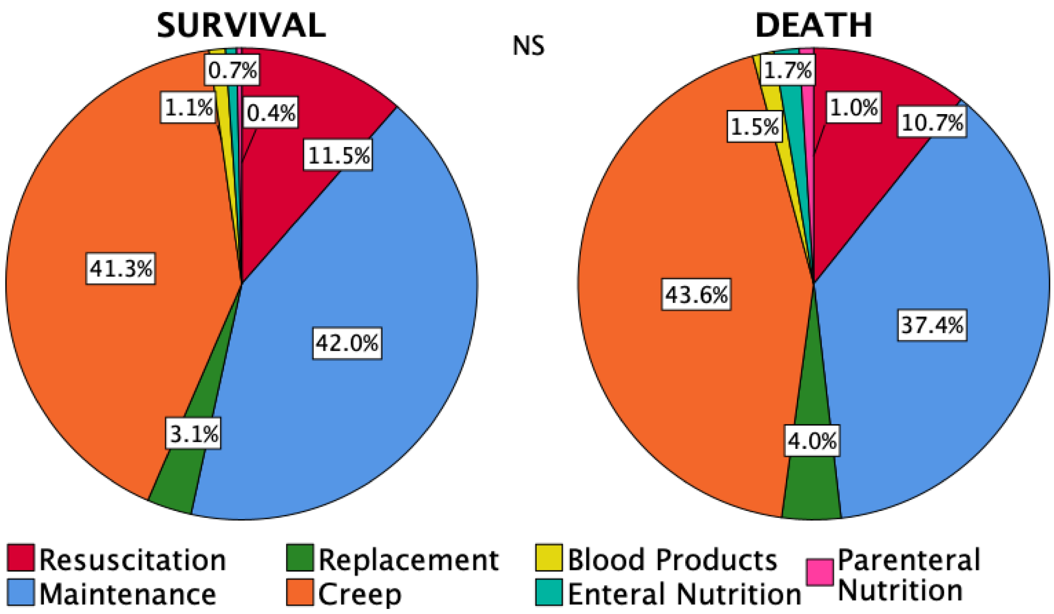

Figure S3. Distribution of cumulative fluid input categories by ICU day 3, indexed to IBW. Fluid creep and maintenance fluids each accounted for >40% of total intake, whereas resuscitation, replacement, and nutrition/blood products contributed smaller proportions.

Table S4. Distribution of cumulative fluid categories by outcome category.

Cumulative Fluids, Days 1-3	Total	Survivors	Non-survivors	p-Value
**Patients, n (%)**	250 (100)	171 (68.4)	79 (31.6)	
**Resuscitation (ml), mean±SD**	1222 ± 896	1198 ± 882	1273 ± 928	0.535
**Resuscitation (ml/IBW), mean±SD**	18.9 ± 13.6	18.4 ± 13.1	20.4 ± 14.7	0.712
**Maintenance (ml), mean±SD**	4334 ± 1175	4316 ± 1206	4375 ± 1111	0.282
**Maintenance (ml/IBW), mean±SD**	68.4 ± 22.7	67.3 ± 23.9	70.9 ± 19.7	0.245
**Replacement (ml), mean±SD**	337 ± 1103	320 ± 1104	374 ± 1116	0.722
**Replacement (ml/IBW), mean±SD**	5.76 ± 19.6	4.9 ± 16.5	7.6 ± 24.9	0.305
**Blood Products (ml), mean±SD**	141.3 ± 705	116.1 ± 627	195.9 ± 851	0.406
**Blood Products (ml/IBW), mean±SD**	2.11 ± 11.1	1.80 ± 10.6	2.78 ± 11.9	0.516
**Enteral Nutrition (ml), mean±SD**	131.1 ± 573	85.2 ± 432	230.2 ± 793	0.063
**Enteral Nutrition (ml/IBW), mean±SD**	1.85 ± 8.11	1.19 ± 6.06	3.28 ± 11.3	0.058
**Parenteral Nutrition (ml), mean±SD**	60.5 ± 438	37.6 ± 359	110.0 ± 572	0.225
**Parenteral Nutrition (ml/IBW), mean±SD**	0.98 ± 6.94	0.56 ± 5.29	1.87 ± 9.56	0.167
**Fluid Creep (ml), mean±SD**	4616 ± 2322	4354 ± 2171	5183 ± 2541	0.008
**Fluid Creep (ml/IBW), mean±SD**	71.3 ± 34.6	66.1 ± 31.6	82.6 ± 38.2	<0.001
**Fluid Creep Plus[a] (ml), mean±SD**	5532 ± 2775	5158 ± 2565	6342 ± 3044	0.002
**Fluid Creep Plus[a] (ml/IBW), mean±SD**	85.4 ± 41.4	78.3 ± 37.9	100.7 ± 44.6	<0.001

[a] *Fluid Creep Plus = Fluid Creep including Blood Products, and Nutrition.*

Supplementary Methods

Supplementary logistic regression analyses were conducted to assess the robustness of associations between fluid variables and ICU mortality. Univariate binary logistic regressions identified individual predictors (Table S5). Variables with $p < 0.10$ or clinical relevance were subsequently included in a multivariable forced-entry model (Table S6). A backward stepwise likelihood-ratio (BSTEP-LR) model was used to derive the most parsimonious set of predictors (Table S7). Collinearity was assessed via variance inflation factors (VIF > 5 = moderate; VIF > 10 = high). Model discrimination and calibration were evaluated using the area under the ROC curve (AUC), Brier score, Cox & Snell R^2, Nagelkerke R^2, and the Hosmer–Lemeshow goodness-of-fit test. Analyses were performed in SPSS v30 (IBM Corp.) and verified in Python 3.12 (statsmodels v0.14).

Table S5. Univariate Logistic Regression for ICU Mortality. Selected variables significantly associated with ICU mortality in univariate logistic regression.

Variable	N	Deaths	OR	95% CI	p-Value
**Fluid overload day 3 (%)**	250	79	1.11	1.06–1.16	<0.001
**Cumulative fluid creep day 3 indexed to IBW**	250	79	1.01	1.01–1.02	0.001
**KDIGO ≥ Stage 2 (yes)**	250	79	2.23	1.19–4.17	0.012
**SOFA score**	249	79	1.11	1.01–1.21	0.034

Abbreviations: OR, odds ratio; CI, confidence interval; IBW, ideal body weight.

Table S6. Multivariable Logistic Regression (Forced-Entry Model).

Variable	Adjusted OR	95% CI	p-Value
**Cumulative fluid creep day 3 indexed to IBW**	1.017	1.003–1.030	0.017
**Fluid overload day 3 (%)**	1.062	1.001–1.126	0.046
**APACHE II**	1.00	0.95–1.06	0.97
**SOFA score**	1.06	0.94–1.20	0.34
**KDIGO ≥ Stage 2**	1.67	0.68–4.09	0.26
**Age**	1.01	0.98–1.03	0.63
**Sex (female)**	0.91	0.43–1.89	0.79

Abbreviations: OR, odds ratio; CI, confidence interval; IBW, ideal body weight.
Model diagnostics: AUC = 0.71; Brier = 0.19.

Table S7. Backward Stepwise (Likelihood-Ratio) Logistic Regression.

Variable	Adjusted OR	95% CI	p-Value
**Cumulative fluid creep day 3 indexed to IBW**	1.020	1.010–1.030	0.012
**Fluid overload day 3 (%)**	1.060	1.010–1.130	0.028

Abbreviations: OR, odds ratio; CI, confidence interval; IBW, ideal body weight.
Model diagnostics: Cox & Snell $R^2 = 0.164$; Nagelkerke $R^2 = 0.229$; Hosmer–Lemeshow $\chi^2 = 12.755$ (df = 8, $p = 0.121$); AUC = 0.95; Brier = 0.07. The model demonstrates good calibration ($p > 0.05$), moderate explanatory power ($R^2 \approx 0.23$), and excellent discrimination.

Table S8. Predictive ability of cumulative fluid variables by day 3, indexed to ideal body weight (IBW) for ICU mortality, based on ROC analysis.

Area Under the ROC Curve

Test Result Variable(s)	**Area**	**Std. Error [a]**	**P-value [b]**	**Asymptotic 95% Confidence Interval**		**Youden's Index**		
				Lower Bound	Upper Bound	Sensitivity	Specificity	Cut-Off
Input D3 (ml/IBW)	0.674	0.041	0.000	0.594	0.754	0.79	0.49	165
FO D3 (%/Day)	0.659	0.045	0.003	0.547	0.725	0.53	0.73	5.9
Fluid Balance D3 (ml/IBW)	0.652	0.045	0.003	0.547	0.725	0.53	0.73	59
Maintenance D3 (ml/IBW)	0.577	0.044	0.004	0.539	0.710	0.54	0.70	68
Creep D3 (ml/IBW)	0.632	0.043	0.004	0.539	0.707	0.72	0.51	66.3

[a] *Under the nonparametric assumption*

[b] *Null hypothesis: true area = 0.5*
